# Expression Profiling of *Plasmodium berghei HSP70* Genes for Generation of Bright Red Fluorescent Parasites

**DOI:** 10.1371/journal.pone.0072771

**Published:** 2013-08-27

**Authors:** Marion Hliscs, Carolin Nahar, Friedrich Frischknecht, Kai Matuschewski

**Affiliations:** 1 Max Planck Institute for Infection Biology, Parasitology Unit, Berlin, Germany; 2 Bio21 Molecular Science and Biotechnology Institute, University of Melbourne, Parkville, Australia; 3 Parasitology, Department of Infectious Diseases, University of Heidelberg Medical School, Heidelberg, Germany; 4 Institute of Biology, Humboldt University, Berlin, Germany; Bernhard Nocht Institute for Tropical Medicine, Germany

## Abstract

Live cell imaging of recombinant malarial parasites encoding fluorescent probes provides critical insights into parasite-host interactions and life cycle progression. In this study, we generated a red fluorescent line of the murine malarial parasite *Plasmodium berghei.* To allow constitutive and abundant expression of the mCherry protein we profiled expression of all members of the *P. berghei* heat shock protein 70 (HSP70) family. We identified *Pb*HSP70/1, an invariant ortholog of *Plasmodium falciparum* HSP70-1, as the protein with the highest expression levels during *Plasmodium* blood, mosquito, and liver infection. Stable allelic insertion of a mCherry expression cassette into the *PbHsp70/1* locus created constitutive red fluorescent *P. berghei* lines, termed *Pb*red. We show that these parasites can be used for live imaging of infected host cells and organs, including hepatocytes, erythrocytes, and whole *Anopheles* mosquitoes. Quantification of the fluorescence intensity of several *Pb*red parasite stages revealed significantly enhanced signal intensities in comparison to GFP expressed under the control of the constitutive EF1alpha promoter. We propose that systematic transcript profiling permits generation of reporter parasites, such as the *Pb*red lines described herein.

## Introduction

Malaria is caused by blood infection of the obligate intracellular parasite *Plasmodium*, single cell eukaryotes that follow a complex developmental program during life cycle progression. Although many aspects of the parasite life cycle are known since decades, previously unrecognized aspects of the clinically silent and diagnostically inaccessible pre-erythrocytic phase, such as cell traversal prior to productive invasion [1], intradermal migration of sporozoites [2], and formation of merosomes as the final step in liver stage maturation [3], have profoundly transformed our understanding of *Plasmodium* biology. These observations were possible because generation of a bright green fluorescent sporozoite line, *CSP::GFP* [4], permitted live imaging of sporozoite-infected animals.

Stable transgenic expression of fluorescent proteins is an established strategy in *Plasmodium* parasites and is an invaluable tool for *in vivo* studies on temporal and spatial gene expression. In addition, fluorescent parasites can serve as reference lines for biological studies. The fluorescent proteins of choice include green fluorescent protein (GFP) from the jellyfish *Aequorea victoria* [4]–[6], red fluorescent protein (RFP) and its improved version, RedStar [7], [8], and the red fluorescent protein mCherry [9]–[11]. The first GFP-expressing *Plasmodium* line was generated by stable integration of a dihydrofolate reductase/thymidylate synthase (DHFR-TS) – GFP fusion protein into the *P. berghei* genome [12]. Due to the low promoter activity, fluorescence was relatively weak, prompting subsequent strategies to search for strong, albeit stage-specific, promoters [4], [8]. This limitation was partially overcome by generation of fluorescent parasites that express GFP under control of the elongation factor 1 alpha (*EF1a)* promoter, resulting in constitutive, but only moderate, fluorescence throughout the parasite life cycle [6].

**Figure 1:**
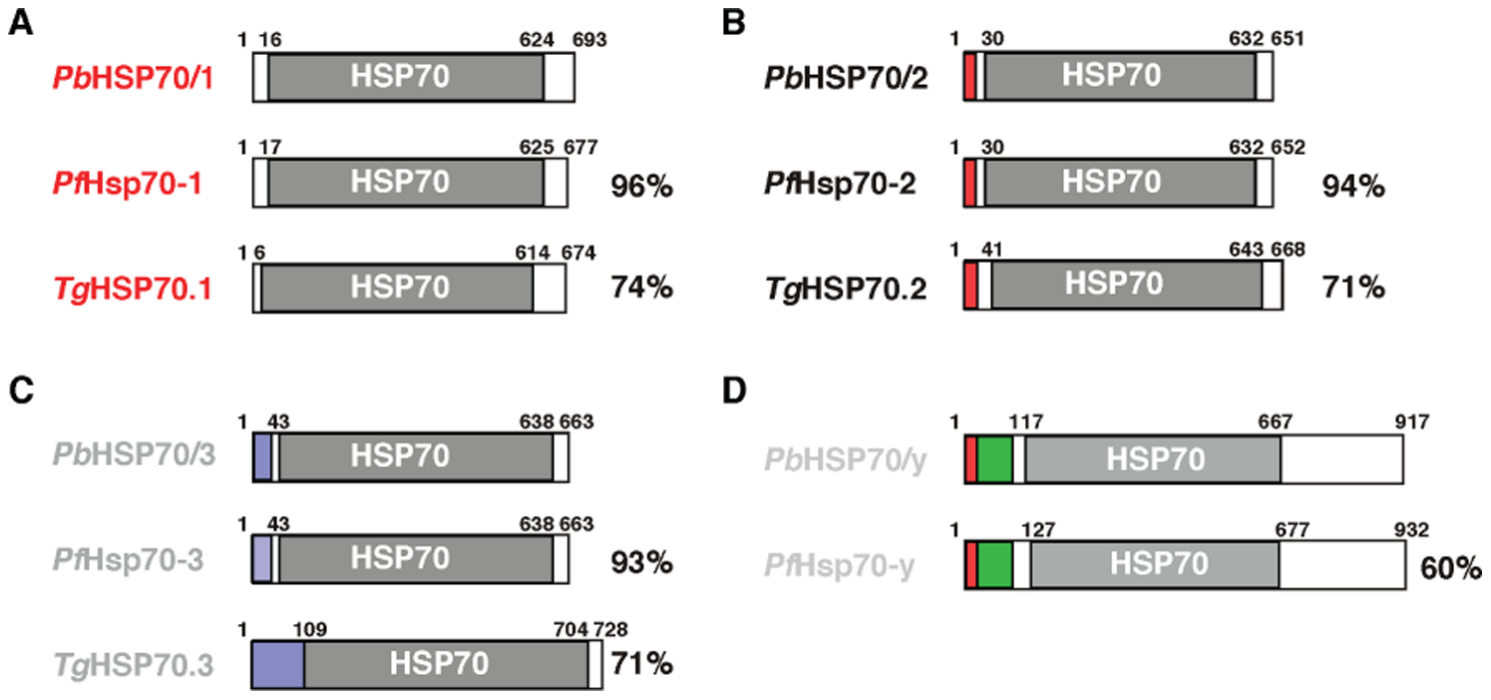
*Plasmodium berghei* heat shock protein 70 (HSP70) proteins. Shown are the primary structures of *P. berghei* HSP70 proteins and their respective *P. falciparum* and *Toxoplasma gondii* orthologs. The signature HSP70 domain (PFAM: PF00012) is boxed in grey. Percentage of amino acid sequence identity of *P. falciparum* and *T. gondii* proteins compared with their *P. berghei* orthologs is indicated to the right. Shown are (A) the cytoplasmic HSC70 proteins *Pb*HSP70/1 (PBANKA_071190), *Pf*Hsp70-1 (PF3D7_0818900; PF08_0054; gi: 23499105), and *Tg*HSP70.1 (TGME49_073760; gi: 3850199); (B) the ER-targeted BiP/GRP78 members *Pb*HSP70/2 (PBANKA_081890), *Pf*Hsp70-2 (PF3D7_0917900; PFI0875w; gi: 124506906), and *Tg*HSP70.2 (TGME49_111720; gi: 237830213) with the cleavable signal peptide boxed in red; (C) the mitochondrial members (mtHsp70/GRP75) *Pb*HSP70/3 (PBANKA_091440), *Pf*Hsp70-3 (PF3D7_113400; PF11_0351; gi: 124804504), and *Tg*HSP70.3 (TGME49_051780; gi: 237836239) with the mitochondrial import sequence boxed in blue; and (D) the candidate apicoplast members *Pb*HSP70/y (PBANKA_135720) and *Pf*Hsp70-y (Pf3D7_1344200; MAL13P1.540; gi: 296005494) with their predicted bipartite targeting sequence boxed in red and green.

In this study, we aimed at generating transgenic, constitutive, red fluorescent *P. berghei* parasites towards robust live cell imaging throughout the entire *Plasmodium* life cycle. For this purpose, we focused on members of the heat shock protein 70 (HSP70) family, because they are ubiquitous, typically abundant, and likely to perform important functions in *Plasmodium* parasites [13]–[15]. In general, HSP70 members are ATPases that tightly bind peptide substrates in their ADP-bound state in order to prevent misfolding or aggregation of the polypeptide substrate, hence the term ‘chaperones’. *Plasmodium* species encode at least four conserved HSP70 members that localize to various compartments in the parasite and likely fulfill distinct chaperone functions (Fig. 1).

The first *P. falciparum* Hsp70 protein to be characterized was the cytoplasmic member, termed *Pf*Hsp70-1 (PF3D7_0818900; PF08_0054; gi: 23499105) [16]–[18]. *Pf*Hsp70-1 contains a distinct carboxy-terminal tetrapeptide repeat, GGMP, which is a signature of the subfamily of heat shock cognate 70 kDa proteins (Hsc70). Importantly, Hsc70 is constitutively expressed, unlike canonical heat shock proteins [19]. The *P. berghei* ortholog (*Pb*HSP70/1; PBANKA_071190) displays an exceptionally high degree of invariant residues [20] (Fig. 1A; Tab. S1). In good agreement, monoclonal antibodies against *Pb*HSP70/1 also recognize recombinant *Pf*Hsp70-1 [21]. A detailed expression analysis using a monoclonal antibody revealed upregulation of *Pf*Hsp70-1 and *Py*HSP70/1 expression in early liver stage development [22]. This finding was subsequently confirmed by the demonstration that *Pb*HSP70/1 is expressed, albeit weak, in sporozoites, lending further support for constitutive expression of this HSP70 member [21]. While the cellular functions of this *Plasmodium* protein have not been addressed experimentally, partial complementation of *Escherichia coli DnaK* [23] and *Saccharomyces cerevisiae SSA1* [24] mutants demonstrated HSP70 activity of *Pf*Hsp70-1. *Pf*Hsp70-1 is a major immunogen of purified blood stage parasites, and an immuno-epidemiological study in a holoendemic area in West Africa revealed an age-dependent acquisition of anti-*Pf*Hsp70-1 antibodies and significant T cell reactivity [25]. Epitope(s) of *Pf*Hsp70-1 are apparently recognized in *Plasmodium*-infected hepatocytes [22] and immunization with recombinant *Py*HSP70/1 leads to an undesired increase in gametocyte formation and, hence, higher malaria transmission [26]. Similarly, a large proportion of sera from *P. vivax* and *P. falciparum*-infected individuals reacted with recombinant *P. vivax* Hsp70-1 [27].

A *Toxoplasma gondii* ortholog, *Tg*HSP70.1 (TGME49_073760; gi: 3850199) was readily identified (Fig. 1A). Intriguingly, posttranscriptional regulation and allelic variants of this protein were implicated in *T. gondii* virulence [28]. Together, these findings indicate that the *HSP70/1* promoter is a strong candidate for transgenic expression of reporter proteins. The structurally related and exported protein *Pf*Hsp70-x (PF3D7_0831700; MAL7P1.228) is present only in *P. falciparum* and the related parasite *P. reichenowi*, where it fulfills a specialized role in remodeling the host erythrocyte [29], and, hence, could not be included in this study (Tab. S1).

Although the two other Hsp70 members are generally less well characterized, they potentially also encode abundant proteins with important functions for life cycle progression (Fig. 1B–C). *Pf*Hsp70-2 (PF3D7_0917900; PFI0875w; gi: 124506906) is translocated to the endoplasmic reticulum (ER) and, hence, resembles binding immunoglobulin protein (BiP)/78 kDa glucose-regulated protein (GRP-78) [30], [31]. It is well conserved among *Plasmodium* species and related apicomplexan parasites (Fig. 1B; Tab. S1). Considerably less work has been done on the mitochondrial Hsp70 (mtHsp70)/75 kDa glucose-regulated protein (GRP-75) proteins encoded by *Pf*HSP70-3 (PF3D7_113400; PF11_0351; gi: 124804504) and its orthologs (Fig. 1C; Tab. S1). The *P. berghei* ortholog *Pb*HSP70/3 (PBANKA_091440) was initially identified as upregulated in infectious sporozoites protein 24 (UIS24), indicative of developmental regulation of *PbHSP70/3/UIS24* expression [32]. A candidate for an apicoplast-targeted Hsp70 member is *Pf*Hsp70-y (Pf3D7_1344200; MAL13P1.540; gi: 296005494) that contains a predicted apicoplast transit peptide in addition to the cleavable signal sequence and a less conserved Hsp70 domain (Fig. 1D; Tab. S1).

We finally included the Hsp70/Hsp90 organizing protein (HOP) in our expression analysis. HOP was originally identified as a 60 kDa stress-related protein that forms a complex with HSP70 and HSP90 [33] and contributes to efficient chaperone cooperation. Initial characterization of *Pf*HOP (PF3D7_1434300; PF14_0324) showed a cytoplasmic localization and indicated that the protein might associate with *Pf*HSP70-1 [34]. *Pb*HOP (PBANKA_101050; gi: 68071103) and *Tg*HOP (TGME49_052220; gi: 237840065) are readily identified based on high sequence conservation (Tab. S1).

In this study, we performed systematic transcript profiling of the *P. berghei HSP70* family throughout the entire *Plasmodium* life cycle and, thereby, identified *Hsp70/1* as a candidate promoter region for robust, constitutive, and high level expression of a red fluorescent reporter protein for live cell imaging applications.

## Results

### Expression Profiling of *P. berghei HSP70* Transcripts

**Figure 2:**
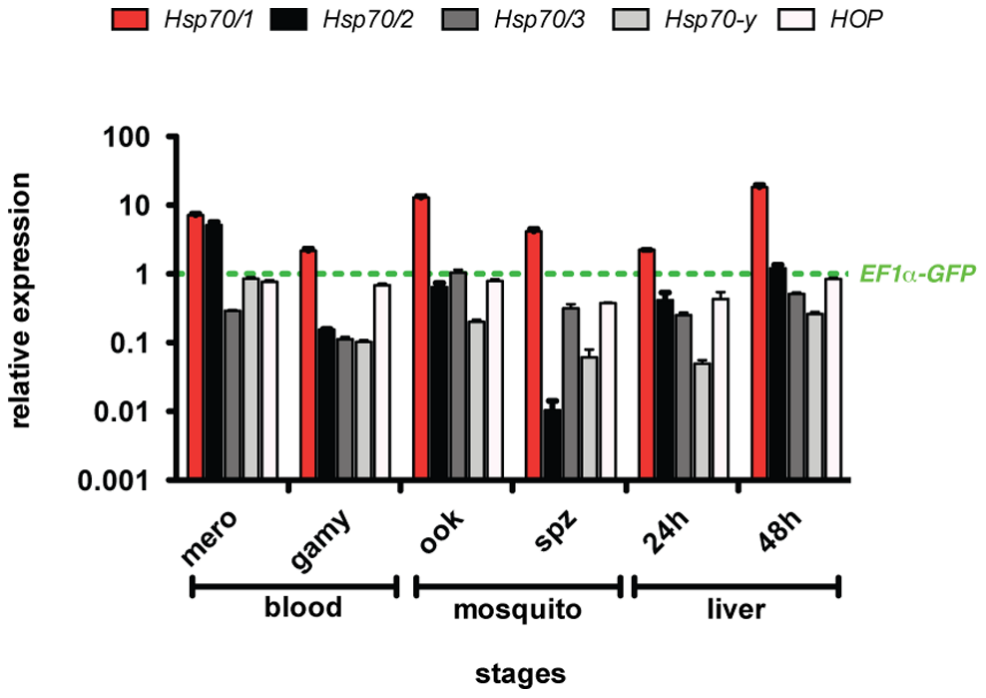
Expression profiling of *Pb*HSP70 members during the *Plasmodium* life cycle. Shown is an expression profiling of *P. berghei* HSP70 mRNAs. *P. berghei* purified schizonts/merozoites (mero), gametocytes (gamy), ookinetes (ook), sporozoites (spz) and 24- and 48-hour liver stages were analyzed by qRT-PCR using primers specific for *Pb*Hsp70/1 (red), *Pb*Hsp70/2 (black), *Pb*Hsp70/3 (dark grey), *Pb*Hsp70-y (grey) and *Pb*HOP (light grey). Expression data from two independent experiments are shown and were normalized to the level of GFP transcripts, which are expressed under the control of the elongation factor 1 alpha (*EF1a*; green dashed line) promoter [6].

We initiated our analysis by systematic quantitative RT PCR (qRT PCR) profiling of *HSP70* and *HOP* mRNAs in different life cycle stages of the murine malarial parasite *P. berghei* (Fig. 2). To this end, we isolated RNAs from (i) gradient-purified late blood stages, so-called schizonts, (ii) enriched gametocytes by drug treatment of *P. berghei*-infected mice, (iii) cultured ookinetes, (iv) sporozoites liberated from infected *Anopheles* salivary glands, and cultured sporozoite-infected hepatoma cells, representing (v) early (24 h) and (vi) late (48 h) liver stages. Profiling of steady-state transcript abundance by qRT PCR using gene-specific primer pairs and normalization to GFP expressed under the control of elongation factor 1 alpha (*EF1a*) revealed robust expression for all transcripts analyzed (Fig. 2).

In all life cycle stages examined, *PbHSP70/1* was the most abundant transcript compared to all other *HSP70* members tested. Expression levels were also typically 2–10-fold higher than the *GFP* reference transcript. This difference was most apparent in ookinetes, schizonts/merozoites, and late liver stages, indicating substantially enhanced signal intensity as compared to *EF1a*, making *PbHSP70/1* the prime candidate to drive reporter gene expression.

*PbHSP70/2/BIP/GRP-78* steady-state levels fluctuated substantially depending on the life cycle phase, ranging from very low (∼100 fold reduced) expression in sporozoites to high (∼10 fold upregulated) levels in schizonts (Fig. 2). The *PbHSP70/2* expression pattern highlights the importance of profiling multiple life cycle stages and indicates differential importance of protein refolding in the ER, and perhaps the organelle as a whole, during *Plasmodium* life cycle progression.

Transcripts of the two organelle-imported HSP70-members, *Pb*HSP70/3 and *Pb*HSP70/y, were stably expressed throughout the life cycle, but never exceeded the levels of the *GFP* reference transcripts (Fig. 2). Finally, *PbHOP* expression resembled *PbHSP70/1* expression, albeit at a substantially lower level (Fig. 2). Together, this analysis identified the *PbHSP70/1* promoter as the best candidate to develop a robust reporter parasite with enhanced fluorescence.

### Generation of *Pb*red Parasites

**Figure 3:**
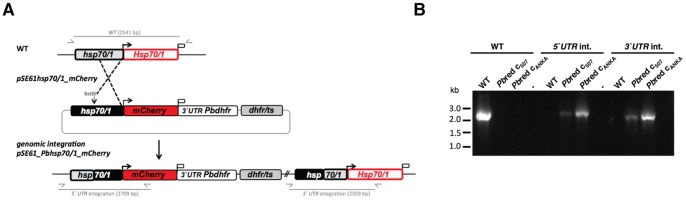
Generation of *Pb*red parasites. (A) Integration strategy to generate *Pb*red parasites. The *PbHSP70/1* genomic locus was targeted with an integration plasmid containing the 5′ region flanking the *HSP70/1* ORF (black box), the mCherry open reading frame (red box) and the 3′ region of the *PbDHFR*, followed by the *Tgdhfr/ts* cassette as positive selectable marker. Upon linearization of the plasmid with *Bst*BI, integration is expected to lead to allele duplication, resulting in mCherry expression under the *PbHSP70/1* promoter and an adjacent *PbHSP70/1* wild-type copy. Integration and wild type-specific test primer combinations and expected fragments are indicated. **(B**) Genotyping of two clonal *Pb*red parasite lines, *Pb*red c_507_ and *Pb*red c_ANKA_. Confirmation of the predicted integration is achieved by PCR analysis using primer combinations (*5′UTR* and *3′ UTR*), which only amplify a signal from the recombinant locus. A wild type-specific primer combination (WT) confirms the absence of residual wild type parasites in the clonal *Pb*red populations.

We next created a *P. berghei* targeting plasmid that would permit stable integration of mCherry under the control of the *PbHSP70/1* promoter (Fig. 3). To this end, we amplified 2.2 kb of the 5′ untranslated region (UTR) of *PbHSP70/1* as the respective promoter sequence and fused it upstream of the mCherry open reading frame, followed by the heterologous 3′UTR of the *P. berghei* dihydrofolate reductase/thymidylate synthase (*DHFR/TS*). Upon linearization in the 5′UTR the targeting plasmid is integrated *via* a single cross-over event (Fig. 3A). The resulting allelic diploid contains a 5′ copy with the desired mCherry expression cassette and a 3′ copy that corresponds to the endogenous *PbHSP70/1* locus.

We performed transfections into two *P. berghei* strains, namely wild-type (WT) parasites (strain ANKA) and clone 507 [6], a WT-like parasite (strain ANKA) that expresses GFP under the control of the constitutive *EF1a* promoter generating *Pbr*ed clone_ANKA_ (*Pb*red c_ANKA_) and *Pbr*ed clone_507_ (*Pb*red c_507_), respectively. Generation of the *Pb*red c_507_ parasite line, emitting GFP controlled by the *EF1a* promoter and mCherry driven under the *Hsp70/1* promoter, allowed us to quantify and compare both fluorescent signals in individual parasites.

Genotyping by diagnostic PCR revealed the predicted site-specific integration in both recipient parasites (Fig. 3B). The resulting parasite lines were cloned *in vivo* by limiting dilutions, resulting in two clonal *Pb*red c_ANKA_ and *Pb*red c_507_ parasite populations.

### *Pb*red Parasites Develop Normally throughout the *Plasmodium* Life Cycle

**Figure 4:**
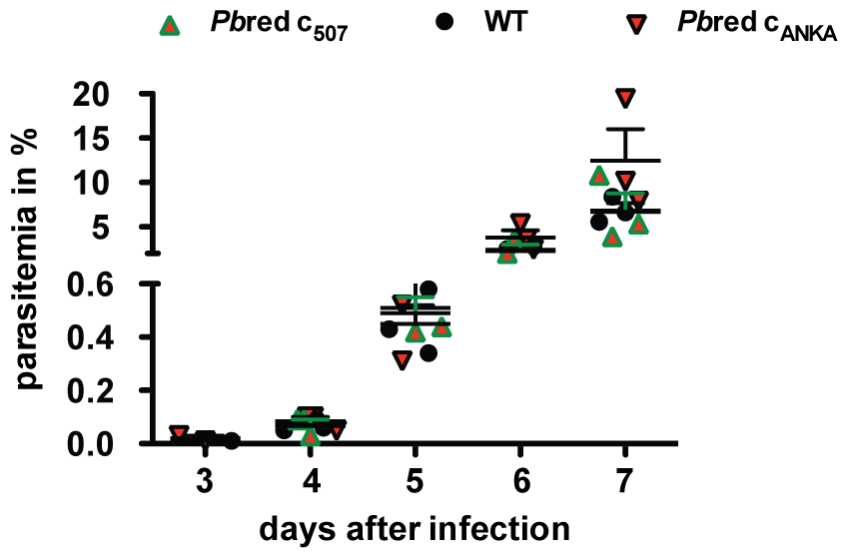
Asexual blood-stage development is unaffected in *Pb*red parasites. Mice were infected intravenously with 1,000 infected erythrocytes. Parasitemia of recipient mice (n = 5 for WT and *Pb*red c_507_; n = 3 for*Pb*red c_ANKA_) was monitored daily by examination of Giemsa-stained blood smears. Shown are mean values (± S.E.M.).

Next, we monitored blood infection in outbred NMRI mice to ascertain that the expression cassette does not interfere with parasite growth *in vivo* (Fig. 4). Monitoring of parasitemia after intravenous injection of 1,000 infected erythrocytes revealed that parasite proliferation and population expansion of both *Pbr*ed clones are indistinguishable from wild-type (WT) parasites during blood stage development.

We next analyzed mosquito stage development of the *Pb*red parasites by determination of salivary gland sporozoite numbers in *infected A. stephensi* mosquitoes (Fig. S1). Sporozoite numbers of *Pb*red c_507_ and WT parasites were indistinguishable, indicating normal life cycle progression in the mosquito host. Furthermore, natural transmission of the *Pb*red c_507_ parasite line was tested by a so-called bite-back experiment. C57/Bl6 mice where exposed to WT- or *Pb*red c_507_-infected mosquitoes and parasitemia was tested daily by microscopic examination of Giemsa-stained blood smears. In all animals tested, blood stage parasites where first detected three days after sporozoite exposure (data not shown). This finding indicates normal liver stage development of *Pb*red c_507_ parasites. Together, we conclude that *Pb*red parasites are suitable as a *P. berghei* reference line, since replication, viability, and infectivity are indistinguishable from WT parasites.

### Live-cell Imaging and Quantitative Fluorescence Analysis of *Pb*red Parasites

**Figure 5:**
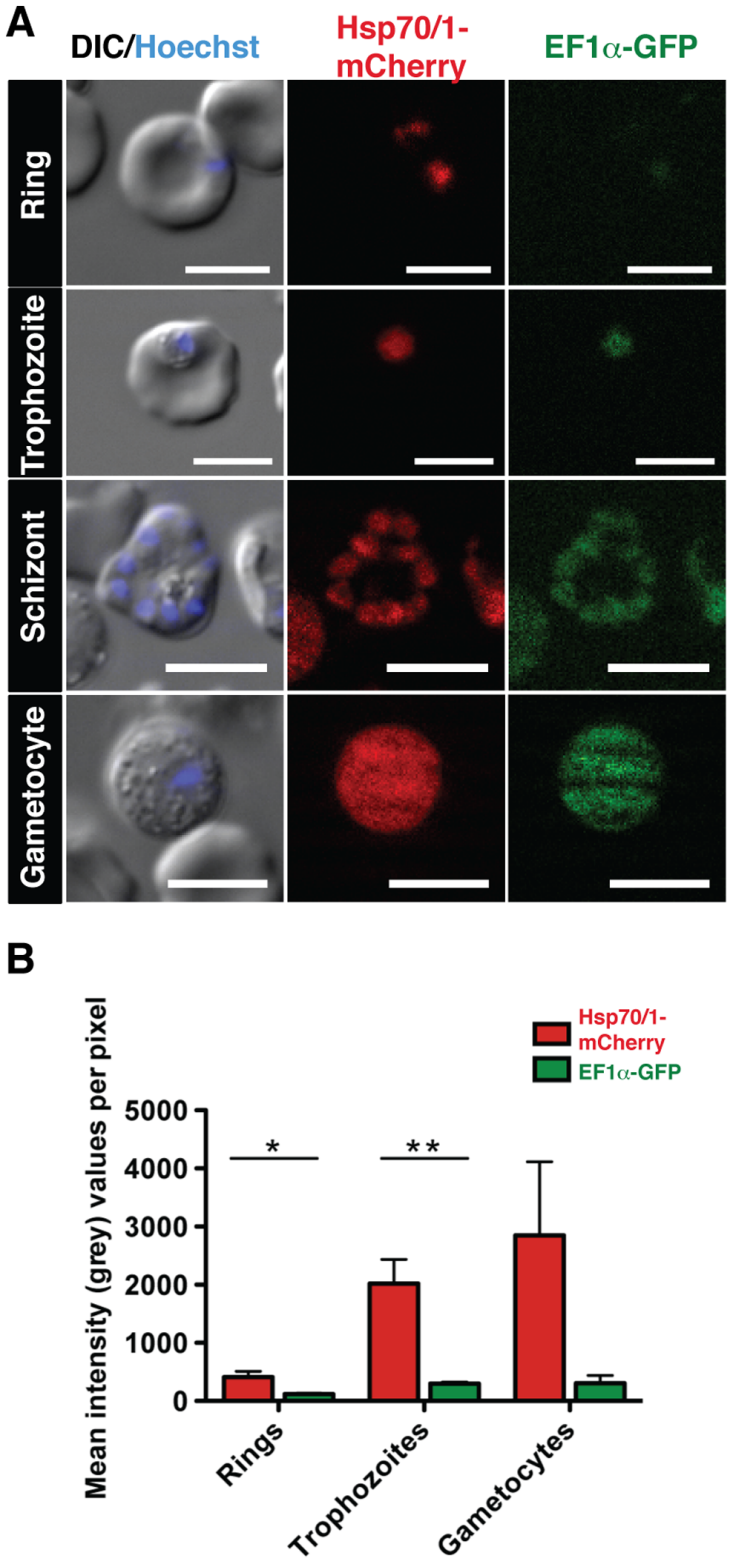
Live imaging and fluorescence quantification of *Pb*red during blood infection. (A) Live cell imaging of *Pb*red c_507_ -infected erythrocytes during blood stage development. Representative differential interference contrast DIC (left) and live fluorescent images for mCherry (center) and the green fluorescent proteins GFP (right) of *Pb*red-infected erythrocytes at different stages of development are shown. Nuclear stains (Hoechst) are visualized as merge in the DIC images. Life cycle stages are indicated on the left. Scale bars, 5 µm. (B) Quantitative analysis of mCherry (red) and GFP (green) fluorescence in different blood stages. Fluorescence intensities are presented in mean of grey values (± S.E.M.) for rings, trophozoites and gametocytes. *, *P*<0.05 (students t-test).

We initiated our imaging analysis with infected erythrocytes (Fig. 5). Fluorescence microscopy of whole blood *ex vivo* revealed bright red fluorescence throughout all asexual blood stage parasites and gametocytes (Fig. 5A). Fluorescence intensity appeared uniform in all stages and permits classical morphological distinction of different parasite stages based on the combination of the strong cytoplasmic red fluorescence and the blue fluorescent nuclear signal obtained with Hoechst 33528. In comparison, the GFP fluorescence signal was relatively low, and virtually undetectable in young parasites, *i.e.* ring stages (Fig. 5A). Quantitative comparison of the mCherry and GFP signals revealed significant differences in ring stages and trophozoites and overall brighter signals for both fluorescent proteins in gametocytes (Fig. 5B).

**Figure 6:**
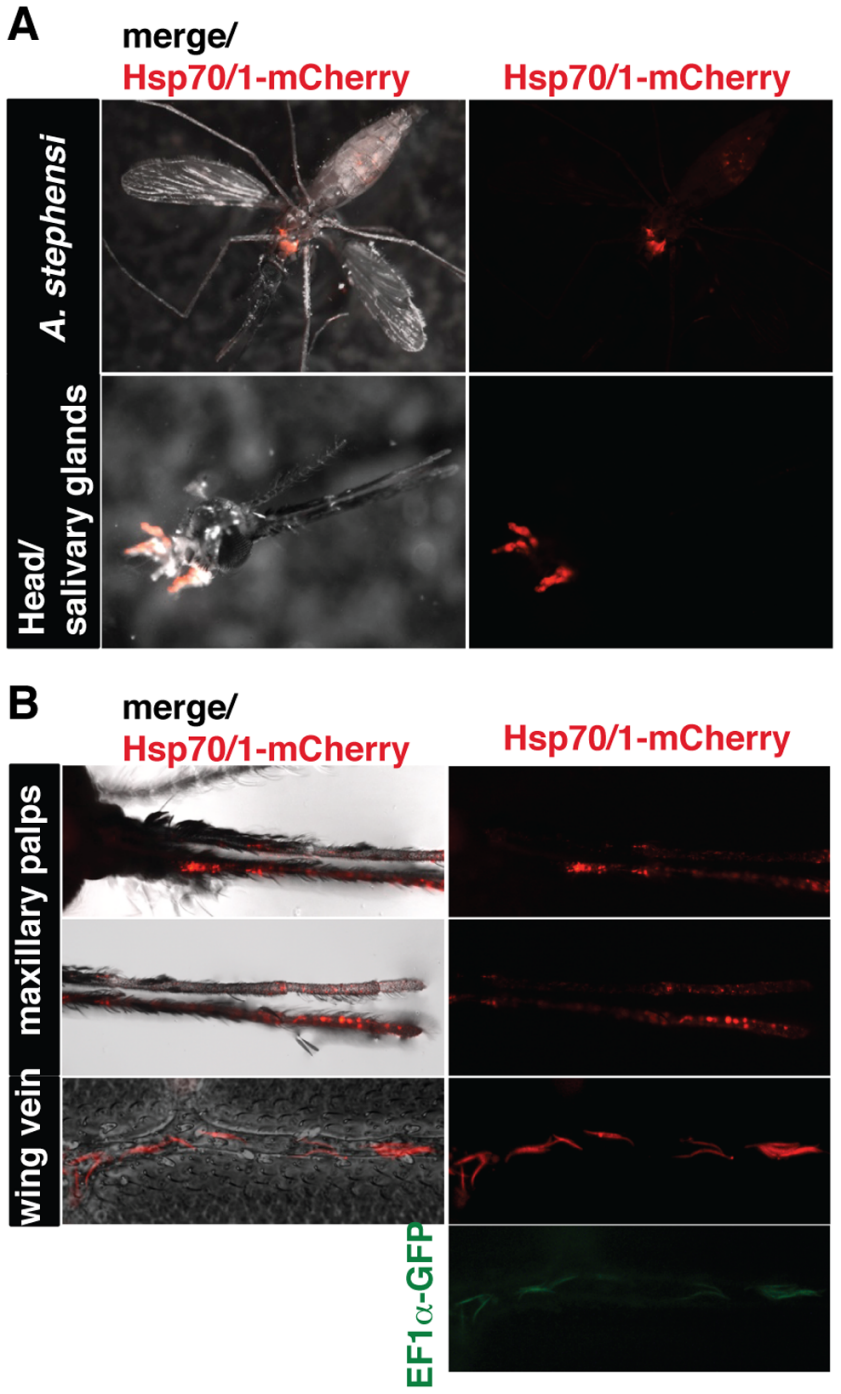
Live imaging of *Pb*red-infected *Anopheles stephensi* mosquitoes. (A) Live imaging of salivary gland colonization of *Pb*red-infected *Anopheles stephensi* mosquitoes. Shown are representative live fluorescent images with the merge of fluorescence and white light illuminated images (left) and the mCherry signal only (right). (**B**) Live imaging of hemocoel sporozoites in *Pb*red-infected *Anopheles stephensi* mosquitoes. Shown are representative higher magnification live fluorescent images of the mosquito maxillary palps (top) and wings (bottom) with the merge of fluorescence and white light illuminated images (left), the mCherry signal (right), and the corresponding GFP signal (bottom right), exemplified in a wing vein.

We next analyzed parasite development in the mosquito vector (Fig. 6). Mosquito infection with *Pb*red c_507_ parasites could readily be identified in the midgut and salivary glands of live mosquitoes, because of the strong red fluorescence of the respective parasite populations in these organs (Fig. 6A). This finding prompted us to explore whether we could image individual sporozoites in whole mosquitoes, as previously achieved with transgenic parasites that express GFP or mCherry under the control of the circumsporozoite protein (*CSP*) promoter [35], [36]. Intriguingly, we could readily capture individual bright red fluorescent hemocoel sporozoites in wing veins and the maxillary palps of infected mosquitoes (Fig. 6B). The corresponding GFP signal was typically too weak for live imaging, as illustrated for hemocoel sporozoites in a wing vein.

**Figure 7:**
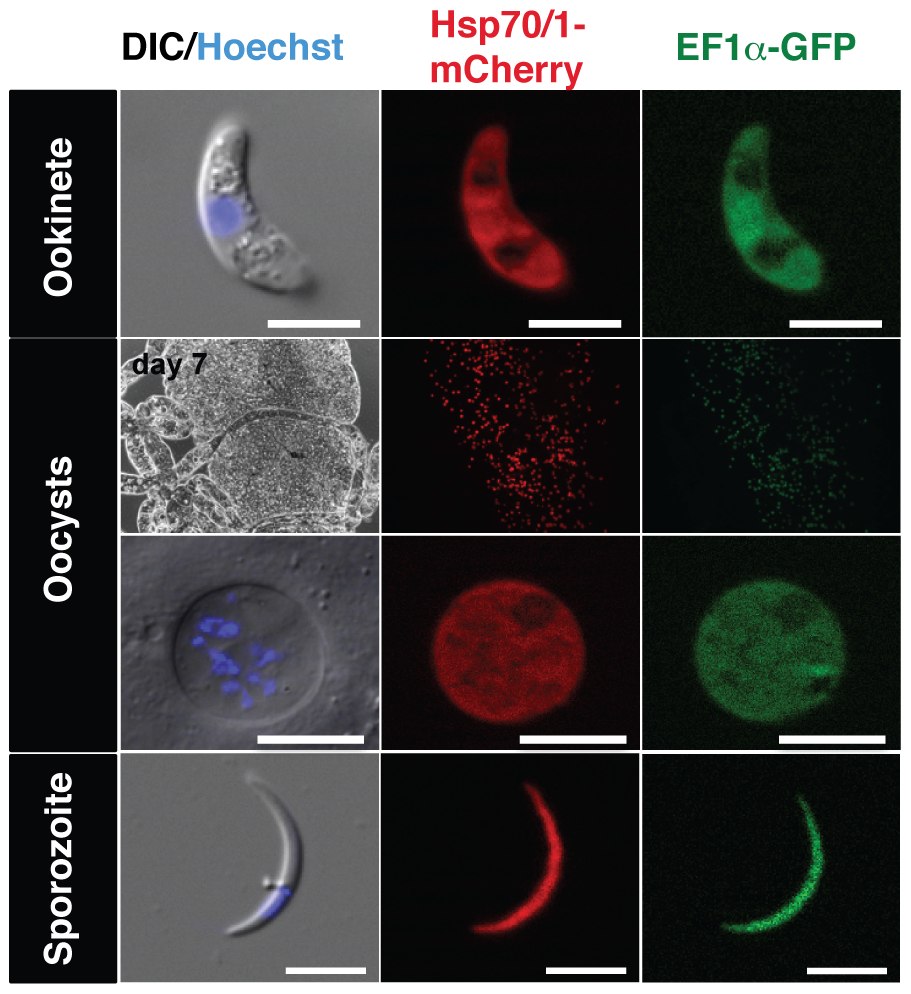
Live cell imaging of *Pb*red during mosquito infection. Representative live cell images of *Pb*red c_507_ development inside the *Anopheles* vector. Presented are DIC images in combination with nuclear stain (Hoechst; left) and fluorescent images for mCherry (center) and GFP (right). Life cycle stages are indicated on the left. Scale bars, 5 µm for ookinete and sporozoite, and 10 µm for oocyst, respectively.

Dissection of *Pb*red-infected mosquitoes at various time points recovered bright red fluorescent ookinetes, infected midguts, single midgut-associated oocysts, and individual salivary gland sporozoites, respectively (Fig. 7). All parasite stages were amenable to live cell imaging and displayed strong red fluorescence signals, as expected from the whole mosquito imaging.

**Figure 8:**
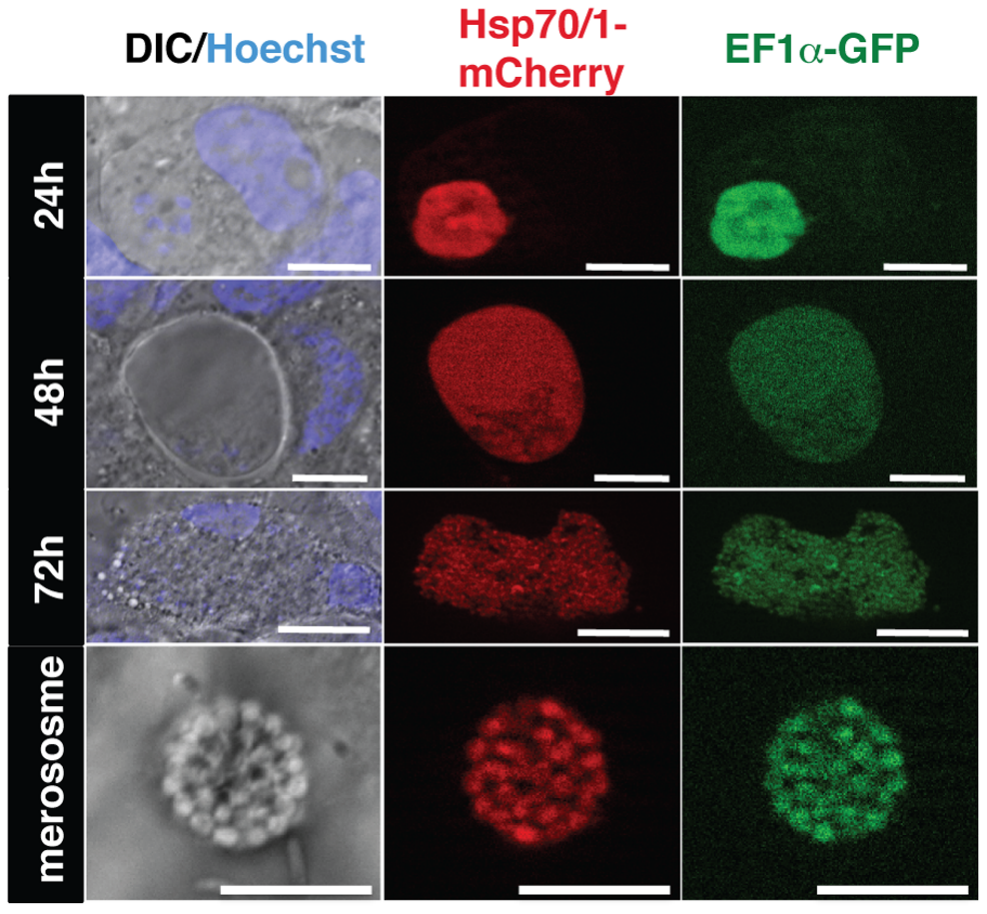
Live cell imaging of *Pb*red parasites during infection of cultured hepatoma cells. Representative live cell images of *Pb*red c_507_ -infected hepatoma cells at different stages of maturation. Presented are DIC images in combination with nuclear stain (Hoechst; left) and fluorescent images for mCherry (center) and GFP (right). Time points after sporozoite infection are indicated on the left. Scale bars, 10 µm for 24 h and 48 h time points and merosomes, and 20 µm for 72 h time points, respectively.

We finally captured the pre-erythrocytic, liver stage development phase of the life cycle (Fig. 8). Intra-hepatic maturation was monitored by *in vitro* infection of human hepatoma cells Huh7 with *Pb*red c_507_ sporozoites. Strong red fluorescence illuminating the parasite cytoplasm of hepatic stages and merosomes, in good agreement with mCherry being a soluble protein that lights up the cytoplasm in all parasite stages described.

**Figure 9:**
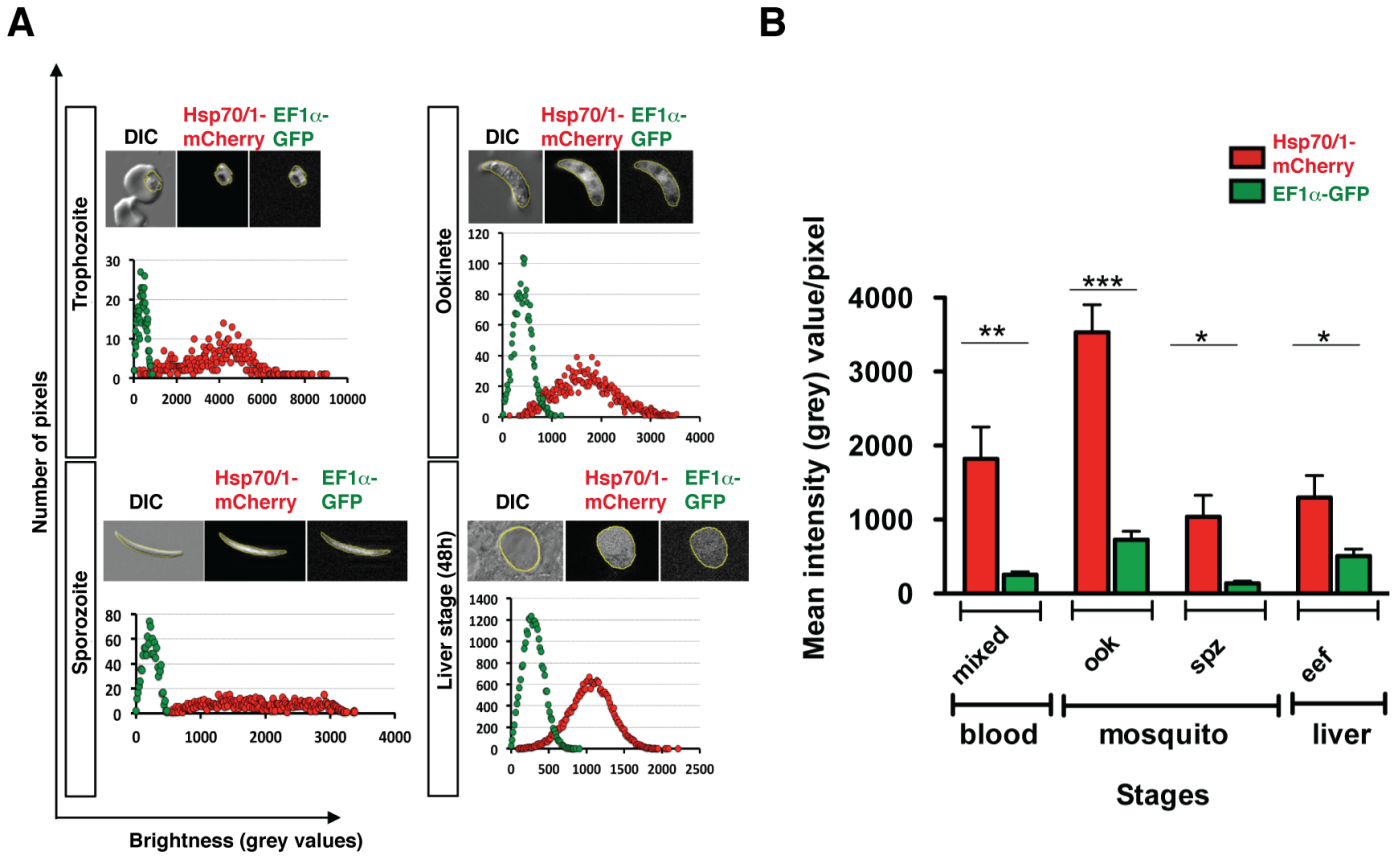
Quantitative analysis of mCherry and GFP fluorescence of *Pb*red c_507_ parasites. (A) Distribution of fluorescence intensities in a representative trophozoite (upper left), ookinete (upper right), salivary gland sporozoite (lower left), and mature liver stage (lower right). Micrographs represent DIC, mCherry and GFP channels with indicated parasite “mask” (yellow), used for fluorescence determination. The point chart displays associated distribution of grey values (brightness) in numbers of pixels for each channel. (B) Quantification of Hsp70/1-mCherry and EF1a-GFP fluorescence in mixed blood stages (n = 12), ookinetes (ook; n = 13), salivary gland sporozoites (spz; n = 4) and extra-erythrocytic liver stages from 24 to 72 h post infection (eef; n = 7). Fluorescence intensities are presented as the mean of grey values for each fluorescent channel (± S.E.M.). *, *P*<0.05; **, *P*<0.01; ***, *P*<0.001 (unpaired students t-test).

In order to compare both fluorophores in a quantitative manner, we performed fluorescence quantification for ookinetes, sporozoites and liver stages of *Pb*red c_507_ parasites (Fig. 9). When we analysed the distribution of the brightness of all pixels for representative parasite stages we detected a broader distribution of high values (Fig. 9A) and significantly higher mean intensities (Fig. 9B) for mCherry as compared to GFP signals in all stages tested.

Together, live cell imaging of the transgene *Pb*red parasite confirmed that mCherry, expression under the Hsp70/1 promoter, results in strong and stable intensity of red fluorescence throughout the entire life cycle as predicted by initial gene expression analysis.

## Discussion

In this study, we developed red fluorescent reporter parasite lines, *Pb*red, termed *Pb*red c_507_ and *Pb*red c_ANKA_, displaying strong and robust fluorescence throughout the entire *Plasmodium* life cycle. We identified a constitutive strong promoter to express mCherry by gene expression analysis of Hsp70 proteins *via* quantitative RT-PCR. This analysis revealed *Pb*Hsp70/1 as the most abundant gene transcript throughout the parasite life cycle, closely resembling constitutive expression of the human ortholog Hsc70. Transcript normalization to GFP expressed under the *EF1a* promoter allowed us to select a promoter that is consistently stronger and most likely appropriate to drive robust and enhanced fluorescence. While we have not addressed *HSP70/1* function by experimental genetics, we consider it highly likely that the gene performs critical roles in all life cycle stages and will be refractory to targeted gene deletion.

All other members of the Hsp70 family, *PbHSP70/2*, *PbHSP70/3*, *PbHSP/y* and *PbHOP* exhibited lower expression levels, in agreement with their organelle targeting or complex organizing role. Intriguingly, expression of the ER-resident *PbHSP70/2* fluctuated substantially. This expression pattern contrasts with the HSP70 members in mitochondria and the apicoplast. Previous work showed that stable fluorescent organelle labeling is feasible [37]–[39]. In agreement with our *PbHSP70/3* and *PbHSP/y* expression data, there was a good correlation of organelle and parasite sizes for apicoplast and mitochondria [38], [39]. It will be interesting to visualize the ER throughout the life cycle and to study whether this organelle expands and contracts, as indicated by the differential expression of *PbHSP70/2.*

The *Pb*red parasite lines were engineered by fusion of mCherry with the *PbHSP70/1* promoter sequence and stable allelic integration by a single crossover event into the *PbHSP70/1* locus. Phenotypic analysis of the *Pb*red clones verified that transgenic expression of the fluorescent protein mCherry did not alter the fitness of the parasite as compared to wild type parasites. We used the double fluorescent *Pb*red c_507_ parasite line to quantify mCherry expressed under the Hsp70/1 promoter with GFP expressed under the *EF1a* promoter [6]. Fluorescence quantitation of diverse *Pb*red c_507_ parasite stages, revealed significant enhanced mCherry signal throughout the life cycle as compared to GFP fluorescence. Our quantification is consistent with earlier reports of bright GFP expression in ookinetes and sporozoites [40], [41]. The quantum yield of GFP (QY = 0.60) and its excitation coefficient (ε = 55,000) are two-fold higher than of mCherry (QY = 0.08, ε = 26,000) [9] (table S3). Nevertheless, the mCherry fluorescence of *Pb*red c_507_ was between three to seven-fold brighter than the GFP signals. This observation is most likely due to the strong promoter activity of *HSP70/1* and is in good agreement with our gene expression analysis. Despite lower intrinsic brightness of mCherry (0.06) as compared to EGFP (1.0) or dtTomato (1.44) advantages of mCherry include fast protein maturation (15 min) and enhanced photo-stability [9]. Accordingly, mCherry is particularly suited for *in vitro* and *in vivo* imaging of *Plasmodium* parasites [11]. Therefore, the *Pb*red clones can be used as reference strains alone or in combination with constitutive green fluorescent parasites [42]. Applications include, but are not limited to, growth competition assays in mice and mosquitoes, cross-fertilization experiments, and establishing kinetics of organ colonization. It is important to note that the expression cassette described herein can also be readily adapted to fluorescent labeling of mutant parasite lines generated by targeted gene deletion [42] and be used for rapid fluorescence-assisted cell sorting (FACS) of recombinant parasites [43]. A recent related example is comparative live cell imaging of wild type and *hsp20(-)* sporozoites, which revealed a specific role for the small heat shock protein 20 (HSP20) in fast, continuous sporozoite locomotion and natural malaria transmission [44].

Fluorescent reporter parasites are important tools to study the complex developmental program, parasite/host cross talk, and parasite population dynamics throughout the *Plasmodium* life cycle. Robust and continuous monitoring of live parasites during processes such as host cell invasion, tissue transmigration, intracellular growth, proliferation, and host cell egress in the mammalian and invertebrate hosts are expected to expand our insights into fundamental aspects of parasite stage conversion and adaptation to new environments. Similarly, capturing of innate defense responses and targeted inactivation by the adaptive immune system will generate important mechanistic insights into the molecular mechanisms of host susceptibility and refractoriness, of naturally acquired immunity, and of vaccine-induced protection. Since effector cells in *Plasmodium* hosts are typically labeled by transgenic GFP expression, mCherry-expressing parasites provide the matching partners for capturing pathogen-host cell interactions at single cell resolution.

## Materials and Methods

### Ethics Statement

All animal work was conducted in accordance with the German ‘Tierschutzgesetz in der Fassung vom 18. Mai 2006 (BGBl. I S. 1207)’, which implements the directive 86/609/EEC from the European Union and the European Convention for the protection of vertebrate animals used for experimental and other scientific purposes. The protocol was approved by the ethics committee of MPI-IB and the Berlin state authorities (LAGeSo Reg# G0469/09).

### Experimental Animals, Parasites, and Cell Lines

Female NMRI and C57Bl/6 mice were from Charles River Laboratories. We used *P. berghei* ANKA and isogenic parasites expressing *GFP* under the control of the *EF1a* promoter [6] as recipient parasites for transfection. Hepatoma cells (HuH7) were cultured as described [10].

### Gene Expression Profiling

For qRT-PCR analyses, mRNA was isolated from schizont-enriched blood stages (merozoites), gametocytes, ookinetes, sporozoites, and 24- and 48-hour liver stages of GFP-expressing *P. berghei* wild-type (WT) parasites (strain ANKA) [6] using the RNeasy kit (Quiagen). After DNase treatment, the mRNA of each sample was reversely transcribed to cDNA using oligo-dT primers of RETROScript kit (Ambion). Quantitative real time RT-PCR was performed on cDNA preparations as described [10] using the StepOnePlus™ Real-Time PCR System and Power SYBR® Green PCR Master Mix (Applied Biosystems) according to the manufacturer’s instructions. qRT PCR was performed in duplicates of one mRNA pool per sample and the whole series was reproduced in a second and third independent experiment. Primers are listed in Table S1. Relative transcript abundance was determined using the 2^−ΔΔCt^ method (Applied Biosystems). Expression data was normalized to the constitutive GFP gene expression of *P. berghei* ANKA GFP parasites [6].

### Generation of Fluorescent *Pb*red Parasites

In order to obtain strong mCherry expression throughout the complete *P. berghei* life cycle, we chose the promoter sequence of the constitutive highly expressed gene *HSP70/1* (PBANKA_071190) protein to drive fluorescent mCherry protein expression. An *HSP70/*1 5′ region of 2,220 bp was selected as promoter sequence and amplified from gDNA using primers HSP70_for and HSP70_rev. The resulting fragment was cloned via *Eco*RI and *Bam*HI restriction sites into a *P. berghei* targeting plasmid, termed pSE61 (kindly provided by Dr. Sabine Engelmann). Subsequently, mCherry was amplified from the plasmid B3D**+**mCherry [10] using primers mCherry_for-BamHI and mCherry_rev_SpeI and cloned via *Bam*HI and *Spe*I downstream of the 5′ promoter sequence. mCherry transcript stability was facilitated by cloning of the 3′ UTR of *P. berghei* dihydrofolate reductase/thymidylate synthase (*DHFR/TS*) downstream of mCherry. The final plasmid, pHSP70/1::mCherry, was used for *P. berghei* transfection [6]. Two independent parasite transfections with clone 507 and clone ANKA GFP were done with 10 µg each of *BstBI*-linearized pHSP70/1::mCherry and gradient-purified schizonts of either green fluorescent ANKA parasites (cl507) [6] or WT ANKA parasites using the Nucleofactor® device (Amaxa). Positive selection of transgene parasites was performed by oral pyrimethamine in the drinking water. Clonal parasite lines were obtained by limiting dilution series and intravenous injection into 6 recipient NMRI mice. Genotyping of clonal parasite lines was performed by diagnostic PCRs using specific primers THsp70_1 for/T16mCherry_rev and THsp70_1rev/pSE61_for detecting 5′ and 3′ integration, in the desired clones, respectively. All primer sequences are listed in Table S2.

### Phenotypical Analysis during the *Plasmodium* Life Cycle *in vivo*

Blood stage development was analyzed *in vivo* in asynchronous infections using NMRI mice. Gametocyte differentiation and exflagellation of microgametes were detected in mice before mosquito feedings. *Anopheles stephensi* mosquito rearing and maintenance was carried out under a 14 h light/10 h dark cycle, 75% humidity and at 28°C. Once infected, *Anopheles* mosquitoes were kept at 80% humidity and 20°C. Sporozoite populations were separated and analyzed as described previously [45]. Briefly, for determination of sporozoite development, mosquitoes were dissected from salivary glands at day 17 after feeding and sporozoite numbers determined. For determination of sporozoite infectivity four C57Bl/6 mice were exposed to 15 *Pb*red and WT infected mosquitoes. Pre patency period was determined by daily microscopic examination of Giemsa-stained blood smears. Asexual blood-stage development was investigated by infection of mice with intravenous injection of 1,000 infected erythrocytes. Parasitemia of recipient mice (n = 5 for *Pb*red c_507_; n = 3 for *Pb*red c_ANKA_) was monitored daily by examination of Giemsa-stained blood smears.

### Live Cell Imaging of *Pb*red c_507_ throughout the *Plasmodium* Life Cycle

Mixed blood stage parasites were obtained by tail vein puncture of infected NMRI mice and imaged live in 37°C pre-warmed RPMI media on ConcavalinA coated glass cover slips. Ookinetes were cultured for 18 h and purified as described [46]. Imaging was done in complete ookinete medium. All mosquito stages were dissected in 3% BSA RPMI media and placed in dissecting media on Vaseline sealed glass slides. Parasite liver stage development was monitored in human hepatoma cells (Huh.7). Cells were infected with 20.000 salivary gland sporozoites in DMEM medium and microscopically observed at indicated time points after infection. Parasite and host cell DNA was visualized with Hoechst 33342 (1∶1,000 dilution; Molecular Probes) for 10 min. Live cell imaging was performed using the Apoptome Imager Z2 Microscope (Zeiss). Representative parasite images where taken using the 20× and 63×objective. Sporozoite colonized proboscis and mosquito wing veins were observed using the 40×objective. Whole mosquito sample pictures were taken with a fluorescent binocular microscope (Leica).

### Quantitative Image Analysis

Live cell imaging of *Pb*red c_507_ parasite stages was performed by wide-field fluorescence microscopy using the Apoptome Imager Z2 Microscope (Zeiss) equipped with an AxioCam (Zeiss). For GFP detection filter sets 38 (Zeiss) with excitation BP 470/40 and emission BP 525/50, and for mCherry detection, filter set 43 (Zeiss) with excitation BP 550/25 were used. Whole *ex vivo* blood samples were imaged in 37°C pre-warmed RPMI media. Images were captured with the Plan-Apochromat 63×/1,40 oil objective (Zeiss), and GFP and mCherry signals were measured using same exposure settings of 20 ms to allow direct comparison. To quantify the fluorescence intensity we determined grey values over the parasite area using the software ImageJ and presented our results as the mean of intensity values per pixel. The distribution of grey values in each channel was measured over the parasite area by drawing a “mask”, encircling the parasite, and the cumulative grey values and corresponding pixel numbers calculated with the histogram function of ImageJ. To compare fluorescence intensities of GFP and mCherry between all parasite stages, the mean of grey values (± S.E.M.) was calculated. Statistical tests were computed with GraphPad Prism 5.0. Statistical significance for fluorescence intensities was determined using the two-tailed t-test. A *P* value of <0.05 was taken as significant.

## Supporting Information

Figure S1 **Normal sporozoite formation of** ***Pb*** **red parasites.** Sporozoite numbers from WT (black; 9,500 (±2,100)) and *Pb*red (striated; 10,300±3,200) parasites where determined by dissection of salivary glands from infected mosquitoes. Sporozoite numbers are depicted as mean (± SEM) from 8 and 11 independent feeding experiments, respectively.
(TIF)

Table S1 Overview of *Plasmodium berghei* Heat Shock Protein 70 (HSP70) members.
(PDF)

Table S2 Primer sequences.
(PDF)

Table S3 Image analysis.
(PDF)
