# Supplementary figures and images for: Expression Profiling of Plasmodium berghei HSP70 Genes for Generation of Bright Red Fluorescent Parasites
Source: PLoS One. 2013 Aug 27;8(8):e72771. doi: 10.1371/journal.pone.0072771 (PMC3754930; doi:10.1371/journal.pone.0072771)

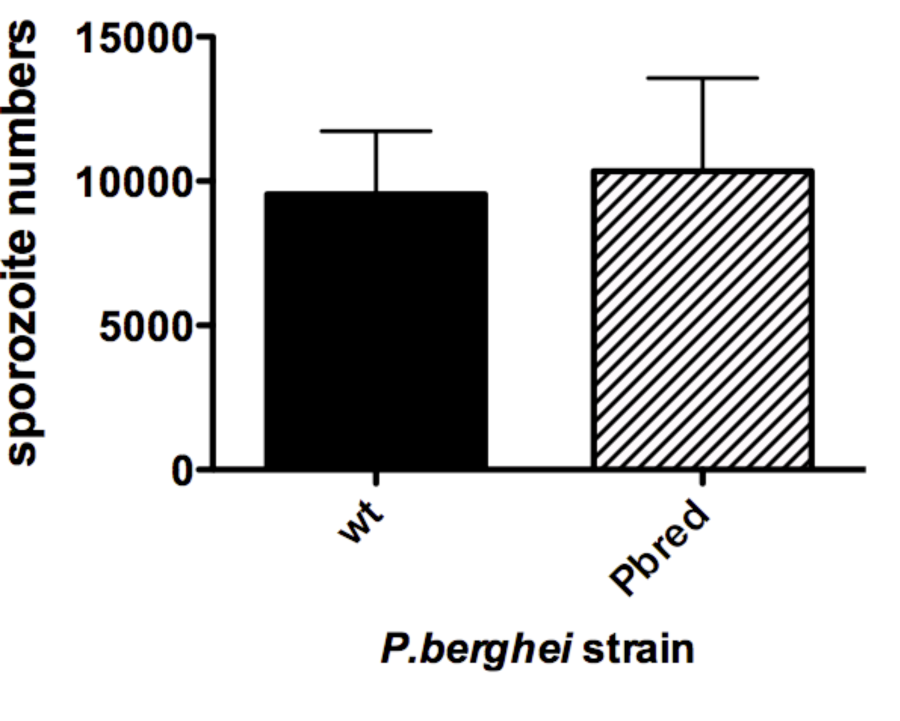

Supplement: Figure S1 — Normal sporozoite formation of Pb red parasites. Sporozoite numbers from WT (black; 9,500 (±2,100)) and Pbred (striated; 10,300±3,200) parasites where determined by dissection of salivary glands from infected mosquitoes. Sporozoite numbers are depicted as mean (± SEM) from 8 and 11 independent feeding experiments, respectively. (TIF) [file pone.0072771.s001.tif]
