# Supplementary material for: Expression Profiling of Plasmodium berghei HSP70 Genes for Generation of Bright Red Fluorescent Parasites
Source: PLoS One. 2013 Aug 27;8(8):e72771. doi: 10.1371/journal.pone.0072771 (PMC3754930; doi:10.1371/journal.pone.0072771)
Supplement: Table S1 — Overview of Plasmodium berghei Heat Shock Protein 70 (HSP70) members. (PDF) [file pone.0072771.s002.pdf]

Supplemental Table S1. Overview of *Plasmodium berghei* Heat Shock Protein 70 (HSP70) members

Pf HSP70[a]	***Pb*HSP70 proteins**				
	***Pb*HSP70/1** (PBANKA_071190)	***Pb*HSP70/2** (PBANKA_081890)	***Pb*HSP70/3/UIS24** (PbANKA_091440)	***Pb*HSP70-y** (PbANKA_135720)	***Pb*HOP** (PbANKA_101050)
***Pf*Hsp70-1** (PF3D7_0818900)	**96%**	54%	43%	21%	**-**
***Pf*HSP70/2/BIP** (PF3D7_0917900)	56%	**94%**	47%	24%	**-**
***Pf*HSP70/3** (PF3D7_1134000)	44%	48%	**92%**	21%	**-**
***Pf*HSP70-y** (PF3D7_1344200)	19%	23%	19%	**59%**	**-**
***Pf*HSP70-x** (PF3D7_0831700)	**-**	**-**	**-**	**-**	**-**
***Pf*HOP** (PF3D7_1434300)	**-**	**-**	**-**	**-**	**83%**

[a] for nomenclature see Shonhai *et al.,* 2007
