# Supplementary material for: Expression Profiling of Plasmodium berghei HSP70 Genes for Generation of Bright Red Fluorescent Parasites
Source: PLoS One. 2013 Aug 27;8(8):e72771. doi: 10.1371/journal.pone.0072771 (PMC3754930; doi:10.1371/journal.pone.0072771)
Supplement: Table S2 — Primer sequences. (PDF) [file pone.0072771.s003.pdf]

Supplemental Table S2. Primer sequences

Gene	Primer Name	Sequence (5' -> 3'; restriction sites underlined)
mCherry	mCherry_for_BamHI	GGGGATCCATGGTGAGCAAGGGCGAGGAGGATAACATGG
	mCherry_rev_SpeI	GCACTAGTCTTGTACAGCTCGTCCATGCC
	T16mCherry_rev	TTCACGTAGGCCTTGGAGCCGTA
GFP	X7_PbGFP_for	GATGGAAGCGTTCAACTAGCAGACC
	X8_PbGFP_rev	AGCTGTTACAAACTCAAGAAGGACC
PbDHFR-TS	pSE_for	CACAAATGATGTTTTTTCCTTCAATTTCGATATCG
PbHSP70/1	X59_Hsp70_4for	GCAAAGCCAAACTTACCAGAGTC
	X60_Hsp70_4rev	CCTTGCAACTTGATTTTTAGCTG
	Hsp70_for	GCGAATTCAATGAGTGAATATGACTTTCATTCG
	Hsp70_rev	GGGGATCCCATGTTTTTTTAATTGTAATTGTAATTTATTGGG
	Thsp1_for	GTATGATTTTTCGTTTCCTTGCAATAGC
	Thsp1_rev	GGTACCCAAATCAATACCTATAGC
PbHSP70/2	X57_Hsp70_3for	TAAGCGCCGTTGACCCAGC
	X58_Hsp70_3rev	GCTTCTAATTTAGCTGCTTCACC
PbHSP70/3	X52_Hsp70_1for	TCTCGCAAAAATCGTTGAAACATGC
	X53_Hsp70_1rev	CGGATGGTGTTGTTCGAAAAC
PbHSP70/y	X54_Hsp70_2for	CAAATTGAATCTTGTAGATAGCG
	X56 Hsp70_2rev	CCGCTGTATATTTTTGCTTCTTC
PbHOP	X61_Hsp70_5for	CTTTATTCAAATTTATCGGGTGCC
	X62_Hsp70_5rev	GGATCTAATTTCAATCCTTCCAAATAA
