# Supplementary material for: Expression Profiling of Plasmodium berghei HSP70 Genes for Generation of Bright Red Fluorescent Parasites
Source: PLoS One. 2013 Aug 27;8(8):e72771. doi: 10.1371/journal.pone.0072771 (PMC3754930; doi:10.1371/journal.pone.0072771)
Supplement: Table S3 — Image analysis. (PDF) [file pone.0072771.s004.pdf]

Hliscs *et al.*,

Supplemental Table S3: Image analysis

**Stage**		**# Number of measured parasites each Channel (gfp/mCherry)**	**Sum**
Blood stages	Rings	3	12
	Trophozoites	6	
	Gametocytes	3	
Ookinetes	(*in vitro* culture)	13	13
Sporozoites	Salivary gland	4	4
Liver stages (EEF)	24 h	1	7
	48 h	3	
	72 h	3	

		Mean grey values (SEM) each stage		Factor	Mean grey values (SEM) each stage ALL (Fig.9B)		Factor
Stage		mCherry	GFP		mCherry	GFP	
Blood stages	Rings	410 ±100	120 ±15	3.4	1,800 ±430	260 ±40	7.1
	Trophozoites	2,000 ±420	300 ±28	6.7			
	Gametocytes	2,850 ±1,300	310 ±130	9.3			
Ookinetes	(*in vitro* culture)	→	→	→	3,530 ±370	730 ±110	4.9
Sporozoites	Salivary gland	→	→	→	1,040 ±300	140 ±30	7.4
Liver stages (EEF)					1,300 ±300	510 ±90	2.6

Supplemental Table S3: Image analysis (continued)

**Fluorophore**<br><br>**Filter set (Zeiss)**	**Excitation (max)**<br><br>**in nm**	**Emission (max)**<br><br>**in nm**
GFP (wt)	396, 475	508
eGFP<br><br>**Filters set 38**	488<br><br>**EX BP 470/40**<br><br>**BS FT 495**	507<br><br>**EM BP 525/50**
mCherry	587	610
Cy3<br><br>**Filter set 43**	(512); 550<br><br>**EX BP 550/25,**<br><br>**BS FT 570**	570; (615)<br><br>**EM BP 605/70**

**Fluorophore**	**Quantum yield QY**	**Extinction coefficient EC (or ε) in $M^{-1}cm^{-1}$**	**Brightness**	**Relative brightness**	**Ref.**
	Relation between the number of photons absorbed to the number of photons emitted	A= εcl<br><br>A absorbance<br>l path of length (cm)<br>c concentration $M^{-1}cm^{-1}$	Brightness= ε*QY	Brightness compared to EGFP<br>(= 01.0)	
EGFP	0.60	55,000	33,000	1.00	Shaner et al., 2004
mCherry	0.22	26,000	2,080	0.06	
